# Alcohol use and depression: link with adherence and viral suppression in adult patients on antiretroviral therapy in rural Lesotho, Southern Africa: a cross-sectional study

**DOI:** 10.1186/s12889-016-3209-4

**Published:** 2016-09-08

**Authors:** Bernard Cerutti, Barbara Broers, Motlomelo Masetsibi, Olatunbosun Faturiyele, Likabelo Toti-Mokoteli, Mokete Motlatsi, Joelle Bader, Thomas Klimkait, Niklaus D Labhardt

**Affiliations:** 1 Faculty of Medicine, University of Geneva, UDREM, 1 Rue Michel Servet, 1211 Geneva 4, Switzerland; 2 Dependencies Unit, Department of Community Medicine, Geneva University Hospitals, Geneva, Switzerland; 3 SolidarMed, Swiss Organization for Health in Africa, Maseru, Lesotho; 4 Department of Biomedicine, University of Basel, Basel, Switzerland; 5 Department of Biostatistics, Epidemiology and Public Health Unit, Swiss Tropical and Public Health Institute, Basel, Switzerland; 6 Clinical Research Unit, Swiss Tropical and Public Health Institute, Basel, Switzerland

**Keywords:** HIV, antiretroviral therapy, Alcohol, Depression, Medication adherence, Africa

## Abstract

**Background:**

Depression and alcohol use disorder have been shown to be associated with poor adherence to antiretroviral therapy (ART). Studies examining their association with viral suppression in rural Africa are, however, scarce.

**Methods:**

This study reports prevalence of depressive symptoms and alcohol use disorder, and their potential association with adherence and viral suppression in adult patients on ART in ten clinics in rural Lesotho, Southern Africa.

**Results:**

Among 1,388 adult patients (69 % women), 80.7 % were alcohol abstinent, 6.3 % were hazardous drinkers (men: 10.7 %, women: 4.4 %, *p* < 0.001). The prevalence of depressive symptoms was 28.8 % (men 20.2 %, women 32.7 %, *p* < 0.001). Both alcohol consumption (adjusted odds-ratio: 2.09, 95 % CI: 1.58-2.77) and alcohol use disorder (2.73, 95 % CI: 1.68-4.42) were significantly associated with poor adherence. There was, however, no significant association with viral suppression.

**Conclusions:**

Whereas the results of this study confirm previously reported association of alcohol use disorder with adherence to ART, there was no association with viral suppression.

**Trial registration:**

April 28th 2014; NCT02126696.

## Background

Despite enormous progress in the roll out of HIV prevention and treatment, 2 million new HIV infections and 1.2 million AIDS-related deaths were reported globally in 2014 with Sub-Saharan Africa bearing the highest burden of morbidity and mortality [1].

For many individuals infected with HIV, the scale-up of antiretroviral therapy (ART) has, however, transformed HIV into a chronic disease that does affect their lives, but does not necessarily shorten their life expectancy, as long as continued adherence is maintained [2, 3]. As HIV has turned into a chronic but manageable condition new challenges arise for the patients and health care providers. Good adherence to ART is particularly decisive for biological and clinical outcomes [4, 5]. Evidence-based recommendations for improving adherence include, among many others, targeting patients with substance abuse and mental illness [6]. Several studies report depression as well as alcohol use disorder to be associated with poor adherence to ART in Africa [7–9]. However, most studies look at self-reported or observed adherence, both prone to reporting-bias. Viral suppression is an observer-independent reflection of drug-adherence and viral load monitoring may be used as an adherence-monitoring tool [10]. Data examining association of depression and alcohol use disorder with viral suppression in Southern Africa are, however, very limited.

Lesotho, an impoverished, landlocked country surrounded by South Africa has the third-highest HIV prevalence in the world [11] and has the highest transmission-rates and the lowest ART-coverage [12] among the hyper-endemic countries in Southern Africa. In this paper we report on prevalence of depressive symptoms and alcohol use, and their potential association with viral suppression in adult patients on ART in rural Lesotho, Southern Africa.

## Methods

### Study setting and participants

The study *Comorbidities and Virologic Outcome Among Patients on Anti-retroviral Therapy in Rural Lesotho* (CART-1) is a registered observational study assessing comorbidities and virologic outcomes among patients on first-line ART in Lesotho (registration: April 28th 2014; NCT02126696). Lesotho is a small landlocked country surrounded by South Africa. It has an adult HIV-prevalence of 23.4 % [13] and on-going high transmission rates [12]. Due to severe shortage of medical personnel, HIV/AIDS care is nurse-based in most clinics. The CART-1 study was conducted in two rural districts, Butha-Buthe and Thaba-Tseka. In each district one hospital and four nurse-led health centres were included. All involved facilities receive support through SolidarMed, a Swiss non-for-profit organization that has been assisting the Ministry of Health in the roll out of ART since 2005. During the CART-1 study, all patients on ART ≥ 6 months attending routine follow-up visits between May 5th, 2014 and June 17th, 2014 and willing to participate received viral load measurement and extensive comorbidity screening, including assessment for alcohol use disorder and depressive symptoms. The study-setting has been described elsewhere [14, 15]. Exclusion criteria were being on ART < 6 months, history of treatment interruption ≥ 7 days during the last 3 months, receiving second-line ART.

For the present analysis, patients aged ≥ 16 years with documented viral load, alcohol use disorder and depressive symptoms screening were included.

### Measures

Participants were enrolled and screened at their routine ART-visits during the study-period. A trained lay-counsellor collected basic socio-demographic data, assessed adherence and subsequently conducted screening for alcohol use disorder and depressive symptoms. The latter were screened for using the Alcohol use Disorder Identification Test (AUDIT) [16] and the nine-item depression module from the Patient Health Questionnaire (PHQ-9) [17] respectively. Both screening tools have been used in similar settings [7–9]. Interviews were conducted in a separate closed room to ensure confidentiality. The internationally recommended thresholds for “hazardous drinker” (AUDIT-score ≥ 8) “alcohol dependence” (AUDIT-score ≥ 20) and depression (PHQ-9 score ≥ 10) were used, as in other studies in Sub-Saharan Africa [18–21]. AUDIT and PHQ-9 questionnaires were translated by a professional English-Sesotho translator and translated back into English by study-personnel. Meaning of questions after back-translation into English was unchanged. Thereafter, the Sesotho questionnaires were discussed in focus groups involving community workers, health care providers and patients where group members agreed on some minor grammar modification.

After the interviews patients attended their routine ART-consultation and blood for biologic analyses was taken. Routine laboratory exams (full blood count, CD4-cell count, serum gamma-glutamyl transferase, and serum creatinine) were performed at the laboratories of the two hospitals involved in the study, which are both certified national laboratories. Blood for the viral load analyses was collected in a Plasma Preparation Tube (PPT), centrifuged within eight hours and subsequently frozen at - 40 °C. PPTs were shipped on dry ice within a maximum of 3 weeks to a reference laboratory in Switzerland. Viral RNA was prepared using an automated extractor (NucliSENS easyMAG, Biomerieux, Switzerland) and quantified using a validated protocol as published [22]. Based on established sensitivity of the protocol, viral suppression was defined as a viral load <80 copies/mL. Adherence was assessed through two different approaches: self-reported adherence using a visual analogue scale (VAS), and pill-count. Low adherence criteria were defined as a VAS-score ≤95 %, or a pill-count <95 %. VAS ≤ 95 % was associated to virologic failure in a previous study in the same setting [23]. Pill-count ≤95 % has been shown to be associated to lower rates of viral suppression in similar settings [24] Participants’ wealth index was assessed using the 2009 Lesotho Demographic Health Survey questionnaire [25], which includes the following variables: type of floor, roof, and walls of the house; type of beds, toilet; material used for cooking; access to water; electricity supply; equipment of the household (electricity generator, radio, tv, fix and mobile phone, watch, fridge, bed, computer, access to Internet, bicycle, cart, motorbike, car); owned surface of land; and a complete description of the owned cattle and sheep.

### Analysis

Unless specified, mean results and odd ratios (OR) are given with 95 % confidence intervals. Link between dichotomous variables and other covariates were investigated with generalized linear models with binomial function links, and the different models were compared with chi square test based on the analysis of deviance. For the prevalence or alcohol use, depressive symptoms, self-reported adherence, and viral suppression, difference between gender were adjusted by age and wealth index. For virologic suppression, the link with the following covariates were investigated: age, sex, wealth index, type of health facility (health centre or hospital), nucleosid reverse transcriptase inhibitor (NRTI) and non-nucleosid-reverse transcriptase inhibitor (NNRTI) of the current ART-regimen, PHQ score, AUDIT score, and self-reported adherence (VAS and pill count). The wealth-index was generated through principal component analysis [26]. Links between two categorical variables were investigated with Chi-squared test. All analyses were run on R 2.15.3 (the R Foundation for Statistical Computing), and TIBCO Spotfire S + ® 8.1 for Windows (TIBCO Software Inc).

## Results

A total of 1,811 adult patients attended ART-consultations during the study-period, 213 (11.8 %) were excluded due to pre-specified exclusion criteria (138 on ART < 6 months, 50 refused participation, 25 on second-line ART) and 35 (1.9 %) due to missing viral load data. Among the 1,563 patients, 1,388 (88.8 %) had both, documented PHQ-9 and AUDIT scores. Main results, stratified by sex, are summarized in Table 1.

**Table 1:** Distribution of age, AUDIT and PHQ scores, treatment adherence, and viral suppression

		Overall *n* = 1,388	Women *n* = 958	Men *n* = 430	Odds-ratio^a^(95 % CI)	*p*-value	Adjusted odds-ratio^b^(95 % CI)	Adjusted *p*-value^b^
Median age (IQR)		43.6 (34.5–53.5)	43.6 (34.5–53.5)	40.8 (32.4–51.4)		<0.001	.	.
Alcohol consumption	Yes	268 (19.3 %)	134 (14.0 %)	134 (31.2 %)	0.35 (0.27-0.47)	<0.001	0.37 (0.28-0.50)	<0.001
	No	1,120 (80.7 %)	824 (86.0 %)	296 (68.8 %)				
Mean AUDIT Score (SD) among drinkers		5.9 (5.4)	5.7 (5.8)	6.2 (4.9)	.	0.454	.	0.491
Hazardous consumption (AUDIT ≥ 8)	Yes	88 (6.3 %)	42 (4.4 %)	46 (10.7 %)	0.38 (0.25-0.59)	<0.001	0.39 (0.25-0.60)	<0.001
	No	1,300 (93.7 %)	916 (95.6 %)	384 (89.3 %)				
Alcohol dependence (AUDIT ≥ 20)	Yes	4 (0.3 %)	3 (0.3 %)	1 (0.2 %)	.	.	.	.
	No	1,384 (99.7 %)	955 (99.7 %)	429 (99.8 %)				
Mean PHQ Score (SD)		6.6 (5.8)	7.1 (5.8)	5.5 (5.6)	.	<0.001	.	<0.001
Moderate to severe depression (PHQ-9 ≥ 10)	Yes	400 (28.8 %)	313 (32.7 %)	87 (20.2 %)	1.89 (1.45-2.49)	<0.001	1.92 (1.45-2.54)	<0.001
	No	988 (71.2 %)	645 (67.3 %)	343 (79.8 %)				
Mean VAS (SD)		92.0 (12.2)	91.9 (12.3)	92.2 (12.0)	.	0.713	.	0.701
Low VAS (≤95 %)	Yes	610 (44.7 %)	419 (44.6 %)	191 (45.0 %)	0.97 (0.77-1.23)	0.823	0.97 (0.76-1.23)	0.780
	No	754 (55.3 %)	521 (55.4 %)	233 (55.0 %)				
Mean pill count Score (SD)		96.9 (8.3)	97.1 (8.1)	96.6 (8.6)	.	0.333	.	0.380
Low pill count (≤95 %)	Yes	301 (22.6 %)	196 (21.3 %)	105 (25.5 %)	0.79 (0.60-1.04)	0.093	0.81 (0.61-1.08)	0.148
	No	1,029 (77.4 %)	723 (78.7 %)	306 (74.5 %)				
Viral Suppression (≤80 copies/m)	Yes	1,270 (91.5 %)	883 (92.2 %)	387(90.0 %)	1.31 (0.88-1.94)	0.187	1.43 (0.96-2.16)	0.088
	No	118 (8.5 %)	75 (7.8 %)	43 (10.0 %)				

^a^the group of reference is the male population

^b^adjusted by age and wealth index

### Alcohol use

The proportion of alcohol abstainers (past 12 months) was 80.7 % (78.6 %-82.8 %; men 68.8 %, 64.4 %-73.2 %; woman 86.0 %, 82.8-88.2 %; *p* < 0.001), and rose from 73.8 % for the lowest quintile of the wealth index to 86.6 % for the highest one (p = 0.002). There were 6.3 % of hazardous drinkers (5.1 %-7.6 %; men 10.7 %, 7.7 %-13.6 %; women 4.4 %, 3.1 %-5.7 %, *p* < 0.001), and 0.3 % of dependent drinkers. Among those who drink, the average AUDIT score was 5.92 (5.27-6.56; men 6.16, 5.33-7.00; women 5.67, 4.68-6.66; *p* = 0.454). The level of gamma-glutamyl transferase correlated significantly with the AUDIT score (correlation of the log values: 0.223; *p* < 0.001).

### Depressive symptoms

The average PHQ-9 score was 6.61 (6.31-6.91; men 5.53, 5.01-6.06; women 7.09, 6.73-7.46; *p* < 0.001), and the prevalence of depression (PHQ-9 score ≥10) was 28.8 % (26.4 %-31.2 %; men 20.2 %, 16.4 %-24.0 %; women 32.7 %, 29.7 %-35.6 %; *p* < 0.001). Depression prevalence decreased from 33.7 % for the lowest quintile of the wealth index to 20.4 % for the highest one (*p* = 0.002).

There was a significant but weak correlation of 0.09 (*p* < 0.001) between AUDIT and PHQ-9 (men: 0.15, women: 0.10), and no evidence of association between PHQ-9 and the NNRTI (efavirenz versus nevirapine) of the current ART regimen (*p* = 0.521):

### Virologic suppression

The rate of patients who did not achieve virologic suppression was 8.5 % (7.0 %-10.0 %; see Table 2 for further details), with no evidence of difference between sex (*p* = 0.158) or wealth index quintiles (*p* = 0.293). Neither depression (Odds ratio (OR) 1.27; 0.82-1.96), nor any alcohol consumption (OR 0.94; 0.58-1.51), nor hazardous alcohol consumption (OR 0.81; 0.39-1.65), showed significant association with unsuppressed viremia. In a complementary analysis, we applied different cut-off values for VAS and pill count suggested by Denison et al. [27] after ROC curve analysis. We also applied different AUDIT cut-off values for the hazardous consumption of alcohol as sometimes suggested (≥7 for males, respectively ≥ 6 for females). In both situations the conclusions remained unchanged.

**Table 2:** Viral suppression and association with socio-demographic characteristics, type of ART-regimen, AUDIT and PHQ scores, and treatment adherence

		Viral load <80 c/mL	≥80 c/mL	Odds-ratio viral suppression (95 % CI)	*p*-value
Male		387 (90.0 %)	43 (10.0 %)	1	
Female		883 (92.2 %)	75 (7.8 %)	1.33 (0.90-2.00)	0.158
Median age (IQR)		43.6 (34.5–53.9)	41.4 (33.2–50.2)		0.181^a^
Age <35 years		334 (90.3 %)	36 (9.7 %)	1	0.329
≥35 years		936 (91.9 %)	82 (8.1 %)	1.23 (0.81-1.86)	
Median wealth index (IQR)		−0.52 (−1.66-1.25)	−0.90 (−1.76-1.13)		0.180^a^
Wealth index	Q1^b^	253 (90.7 %)	26 (9.3 %)	1	0.293
	Q2	249 (88.9 %)	31 (11.1 %)	0.83 (0.41-1.64)	
	Q3	250 (92.6 %)	20 (7.4 %)	1.28 (0.60-2.75)	
	Q4	258 (93.8 %)	17 (6.2 %)	1.56 (0.70-3.46)	
	Q5	260 (91.5 %)	24 (8.5 %)	1.11 (0.54-2.31)	
Type of facility	Center	768 (91.9 %)	68 (8.1 %)	1	0.547
	Hospital	502 (90.9 %)	50 (9.1 %)	0.89 (0.61-1.30)	
NRTI backbone	AZT^c^/3TC^d^	418 (87.8 %)	58 (12.2 %)	0.52 (0.34-0.80)	0.004
	TDF^e^/3TC^d^	819 (93.3 %)	59 (6.7 %)	1	
	ABC^f^/3TC^d^	13 (92.9 %)	1 (7.1 %)	0.94 (0.09-9.80)	
NNRTI	EFV^g^	959 (92.2 %)	81 (7.8 %)	1	0.056
	NVP^h^	291 (88.7 %)	37 (11.3 %)	0.66 (0.44-1.00)	
Median AUDIT Score (IQR)		0 (0–0)	0 (0–1.0)		0.384^a^
Hazardous consumption	Yes	79 (89.8 %)	9 (10.2 %)	0.80 (0.39-1.65)	0.559
(AUDIT ≥ 8)	No	1’191(91.6 %)	109(8.4 %)	1	
Median PHQ-9 Score (IQR)		6 (1–10)	6 (1–9)		0.423^a^
Moderate to severe depression (PHQ-9 ≥ 10)	Yes	371 (92.8 %)	29 (7.3 %)	1.27 (0.82-1.96)	0.281
	No	899 (91.0 %)	89 (9.0 %)	1	
VAS (IQR)		96 (91–99)	96 (90–100)		0.424^a^
Low adherence VAS (≤95 %)	Yes	558 (91.5 %)	52 (8.5 %)	1.02 (0.70-1.50)	0.818
	No	691 (91.6 %)	63 (8.4 %)	1	
Pill count’s tool (IQR)		100 (96–100)	100 (96–100)		0.851^a^
Low pill count’s tool (≤95 %)	Yes	275 (91.4 %)	26 (8.6 %)	0.99 (0.63-1.56)	0.999
	No	941 (91.4 %)	88 (8.6 %)	1	

^a^: Wilcoxon rank-sum test, ^b^: lowest quintile, ^c^AZT: zidovudine, ^d^3TC: lamivudine, ^e^TDF: tenofovir, ^f^ABC: abacavir, ^g^: efavirenz, ^h^: nevirapine

### Adherence

The median adherence measured with the VAS was 96 % (interquartile range (IQR): 91 %-99 %), with no evidence of difference between sex (*p* = 0.713). The prevalence of VAS ≤ 95 % was 44.7 % (*p* = 0.823 for difference between sex), decreased from 22.6 % for the lowest quintile of the wealth index to 16.2 % for the highest one (*p* < 0.001), and did not show evidence of association with depressive symptoms (*p* = 0.386). Hazardous alcohol consumption increased the probability of VAS ≤ 95 % (OR 2.89; 1.79-4.66; adjusted by wealth index, type of facility, depressive symptoms: 2.73, 1.68-4.42). For alcohol consumption, these results were 2.19 (1.67-2.90) and 2.09 (1.58-2.77) respectively.

The median adherence measured by pill count was 100 % (IQR 96 %-100 %), with some evidence of lower adherence among men (99 % vs. 100 %; *p* = 0.036). The prevalence of pill count ≤ 95 % was 22.6 % (*p* = 0.093 for difference between sex), and did not show evidence of association with depressive symptoms (*p* = 0.725), Hazardous alcohol consumption increased the probability of low pill count (1.77, 1.08-2.89; adjusted by type of facility and age: 1.68; 1.03-2.75). For alcohol consumption, these results were respectively 1.48 (1.09-2.02) and 1.38 (1.01-1.89).

## Discussion

### Main findings

This cross-sectional study is among the first reporting on prevalence of depressive symptoms and hazardous alcohol use among adult patients on ART investigating their association with unsuppressed viremia in rural Southern Africa. In line with other studies conducted in similar settings, we found hazardous alcohol use to be associated with low self-reported (VAS) adherence to ART [28, 29] or pill count. In addition we also found and association between adherence and alcohol consumption. However, neither hazardous alcohol consumption nor depressive symptoms appeared to be associated with viral suppression through ART.

As an observational cross-sectional facility-based study, interpretation of the results should be limited to patients retained on ART ≥ 6 months. Our study population mainly involved women (69.0 %). This is however representative for the gender-distribution in patients enrolled on ART in Lesotho [15].

### Alcohol consumption

Overall alcohol-consumption and rate of hazardous alcohol use were lower than in a recently conducted survey in the general population [28, 29], and are negatively correlated with the wealth index. The significant association between the level of gamma-glutamyl transferase and the AUDIT confirmed the validity of the instrument in this setting. The proportion of alcohol abstainers in our study-population (68.8 % for men, and 86.0 % for women respectively), was similar [30] or higher [31] to the one observed in other studies. Alcohol abstinence was significantly higher than in the general adult population of Lesotho [32] (58.0 % for men and 81.3 % for women). One explanation may be that in general ART-providers at facilities involved advocate for alcohol abstinence when a patient decides starting ART.

The amplitude of the effect of hazardous alcohol consumption on low adherence (adjusted OR 2.72) is higher to the one found in the literature: a meta-analysis published by Hendershot and colleagues in 2009 reported an OR of 1.82 [33]; Denison et al. report a pooled OR of 1.87 [27], and Ayay et al. 2.17 [30]. However, these studies did not examine for association with viral suppression.

### Depressive symptoms

In contrast to alcohol consumption, depressive symptoms (31.7 % of women and 20.2 % of men) showed a considerably higher prevalence than the reported 10 % in the general population [34]. There was also a negative association of depressive symptoms with the wealth index. This outcome is similar to the one of the systematic review done by Nakimuli-Mpungu et al. in 2012 [35], with a prevalence of 31.2 %. Our study showed no association of depressive symptoms with viral suppression. This finding is in contrast to the study of Marconi et al. reporting association of depression to unsuppressed viremia in a case–control study [36]. The stronger association between AUDIT- and PHQ-9-score among men in our study suggests that particularly among men alcohol consumption should be addressed in cases where of symptoms of depression are found.

### Limitations

This study has several limitations: first, it was carried out in two rural districts of Lesotho, and may not be representative of the situation in the whole country – in particular as it does not involve urban areas. Also, whereas the design of CART-1 could reduce bias when compared to a case control study, the hypothesis of biases cannot be ruled out: many variables collected through questionnaires require recall of past events by the participant. Another limitation is the potential of distortion affecting the association between viral suppression and self-reported adherence due the negative cognitive bias in people with depressive symptoms. Moreover, the study involved only patients on ART ≥ 6 months attending routine follow-up visits: prevalence of alcohol use, depressive symptoms, reported adherence is likely to be different among those patients who have been lost to follow-up. Also PHQ-9 and AUDIT were used at the time of enrolment, which did not allow the study of their evolution in time. Finally we were not able to perform multivariate analysis to adjust for confounding when investigating unsupressed viral load due to the small number of patients with viral load <80 c/mL.

Internationally recommended cut-offs for PHQ-9 and AUDIT were used in this study. PHQ-9 has been used to screen HIV-infected persons for depression in several settings in Sub-Sahara Africa [9, 37–40]. Two independent studies, one from Ethiopia and one from Uganda concluded that a score ≥10 was most accurate to diagnose moderate to severe depression [19, 20]. Nevertheless, PHQ-9 and the used cut-off of 10 for diagnosis of depression has never been validated in Lesotho specifically. Similarly, AUDIT and the cut-offs applied in our study were never used in Lesotho. There are, however, several studies validating AUDIT and the cut-off of ≥8 and ≥20 for “hazardous alcohol use” and “dependence” in similar settings of neighbouring South Africa [21, 41, 42].

## Conclusions

A worth reporting finding in our study is that contrarily to many other publications neither self-reported adherence nor pill-count were associated to viral suppression. Whereas in study settings with well-trained health care providers pill-count and self-reported adherence showed to be predictive for virolologic outcomes, they appear to be imprecise in the setting our study was conducted [24, 43]. Similarly Haberer et al. report from a study in Uganda where pill-count and self-reported adherence showed poor correlation to virologic outcomes [44]. With the expected roll-out of viral load technologies in resource-limited settings, routine viral load monitoring – a more objective adherence measurement - may replace traditional adherence measurement in future [10].

In summary, prevalence of depressive symptoms was generally high and increased inversely to household wealth in this population of ART-patients on rural Lesotho, but no association to virologic outcomes could be shown. Prevalence of hazardous alcohol use was lower than in the general population and increased inversely to household wealth. Prevalence among females was very different than among males, underlining the importance of gender-disaggregated data. Hazardous drinking was associated to poor adherence, but not to unsuppressed viral load.

This study underlines the need for integration of mental health care into ART-services. Health care providers should be encouraged to address with their patients alcohol use and depressive symptoms.

## Abbreviations

3TC, lamivudine; ABC, abacavir; ART, antiretroviral therapy; AUDIT, alcohol use disorder identification test; AZT, zidovudine; EFV, efavirenz; EKNZ, ethics committee of North-West and central Switzerland; IQR, interquartile range; NNRTI, non-nucleosid-reverse transcriptase inhibitor; NRTI, nucleosid reverse transcriptase; NVP, nevirapine; OR, odds ratio; PHQ, nine-item depression on the patient health questionnaire; PPT, plasma preparation tube; ROC, receiver operating characteristic; SD, standard deviation; TDF, tenofovir; VAS, visual analogue scale
